# Neuroplasticity and Functional Recovery after Intensive Language Therapy in Chronic Post Stroke Aphasia: Which Factors Are Relevant?

**DOI:** 10.3389/fnhum.2017.00332

**Published:** 2017-06-28

**Authors:** Bettina Mohr

**Affiliations:** Department of Psychiatry, Campus Benjamin Franklin, Charité Universitätsmedizin BerlinBerlin, Germany

**Keywords:** aphasia, brain reorganization, hemispheres, intensive language therapy, neuroplasticity

## The relevance of neuroscience-based treatment for post stroke aphasia

Neuroscience-based interventions for aphasia are among the most promising approaches toward successful language rehabilitation. Associated therapeutic techniques are highly effective in reducing cognitive-behavioral difficulties resulting from brain damage and can induce neuroplasticity (Taub et al., 2002; Berthier and Pulvermüller, 2011). However, it is still not clear how language recovery and reorganization of language is reflected by functional changes manifest in human brain activity. In aphasia research, previous studies addressed the role of the left (LH) and right (RH) hemisphere across the entire recovery phase. It has been suggested that brain reorganization in aphasia is a dynamic process, in which the involvement of perilesional LH regions and RH areas (e.g., Broca-homolog or RH superior temporal cortex, Musso et al., 1999) is dependent on the specific phase of language recovery (Saur et al., 2006). These data highlight the importance to control for time post stroke when investigating treatment-related changes to avoid the influence of spontaneous remission.

To date, only few studies have addressed therapy-induced neuroplasticity in patients with post stroke aphasia (PSA), and findings are rather inconsistent. The reasons for the heterogeneity of previous results may be attributed to patient-specific variables such as lesion size and site, symptom severity, etc. (Crosson et al., 2005). While patient-specific variables seem to influence functional recovery, the specific impact of non-patient factors (e.g., neuroimaging method, language task, therapy method,—intensity,—amount, and—duration) has rarely been addressed. However, to maximize therapy outcome, it is important to systematically investigate all possible factors and to identify neurophysiological predictors of recovery. Focusing on intensive, clinically effective, and short-term interventions and on suitable language tasks seems promising to achieve this goal.

## Understanding the neuronal mechanisms underlying language recovery by studying treatment-induced changes

Measuring neuronal correlates of good (or poor) language recovery could help to identify patients who might benefit (or not) from a specific therapeutic method and can contribute to the development of effective neuroscience-based neurorehabilitation techniques. The study of *therapy-induced neuroplasticity* in PSA offers the possibility to systematically control for potentially confounding variables while at the same time measuring behavioral and neuronal changes following speech and language therapy (SLT) within a reasonable time frame. Previous findings do not allow to determine whether any behavioral or neurophysiological changes were caused by a specific therapeutic technique or by other factors. This differentiation is only possible by randomized controlled trials (RCTs) with chronic patients (>1 year post stroke), for whom spontaneous remission effects can be excluded. Only RCTs with at least two active treatment groups and a no-treatment waiting-list group will ultimately allow to determine the specificity and amount of therapy-induced changes. Additionally, multiple pre-, and post-therapy scanning sessions will help to control for repeated scanning effects (Fridriksson et al., 2006). Although none of the clinical RCTs conducted in chronic aphasia comprised measurement of brain correlates before and after therapy, a promising heuristic is provided by efficient intensive interventions in chronic PSA patients. In fact, some intensive interventions have proved to lead to significant and consistent improvements of language performance (Brady et al., 2016; Breitenstein et al., 2017) and may therefore be good candidates to study neuroplasticity.

## Neuroplasticity after intensive aphasia treatment methods

Within a short period of time (for example <4 weeks), spontaneous, non-therapy-related neuronal changes are highly unlikely in chronic patients, therefore, one can interpret any changes in behavior and brain activity across short-term intervals as treatment-induced. While intensive training can lead to such changes already within days or weeks (see Berthier and Pulvermüller, 2011), these effects are unlikely for non-intensive methods. In fact, several RCTs demonstrated higher efficacy of intensive SLT compared to non-intensive treatment (Brady et al., 2016). Although it is not entirely clear yet, which treatment intensity and duration is required to induce optimal recovery, meta-analyses suggest a minimum of 1–2 h a day over a period of 2–4 weeks (Bhogal et al., 2003). This therapy frequency might be regarded as *intensive* treatment.

The efficacy of intensive therapies can be explained by neuroscientific principles of learning and memory: high intensity and massed practice facilitates and enhances learning and cortical plasticity by correlated neuronal activity and by strengthening of synaptic contacts between neurons (Berthier and Pulvermüller, 2011). From this perspective, any intensive cognitive-behavioral intervention can maximize the effects of training-induced brain plasticity, which is ultimately a consequence of effective learning. Similarly, multiple repetitions of stimuli or tasks applied during language training, as well as the imitation of language skills modeled by language therapists will enhance learning and re-structuring of residual language networks. However, it should be noted that intensity, repetition, or inter-individual patient characteristics are not the only factors impacting on learning and language recovery. For example, a recent cross-over RCT demonstrated a significant influence of the therapy method used: Communicative-pragmatic and behaviorally relevant language training in social interactions resulted in significantly better recovery than equally intensive conventional exercises such as naming and describing pictures (Stahl et al., 2016). The importance of behavioral relevance and effective neurorehabilitation techniques for functional outcome has also been suggested in the context of motor deficits caused by stroke (e.g., Taub et al., 2002). Behaviorally relevant therapeutic methods delivered at high intensity could provide an avenue toward effective treatment in chronic PSA and may help to increase motivation, engagement and compliance of patients.

## Neuroplastic changes in the left and right hemisphere following intensive short-term aphasia therapy

A recent review (Crinion and Leff, 2015) of neuroplastic changes following therapy in PSA patients, reported consistent treatment-related brain activation changes in LH perilesional fronto-temporal regions and/or in the inferior frontal gyrus in the RH. Unfortunately, due to the heterogeneity of patient and non-patient variables in these intervention studies, it is difficult to draw any concise conclusions about the neuronal mechanisms underlying language recovery. Several factors might explain this variability of previous findings and could be relevant for interpreting treatment-related data and for identifying predictors of recovery. These factors include the phase of PSA (see Saur et al., 2006), symptom severity (Lazar et al., 2010), or education and cognitive reserve (Hillis and Tippett, 2014). Also, the degree of premorbid functional lateralization of language could influence functional recovery (Knecht et al., 2002), as well the type of aphasia. Interestingly, language recovery does not seem to be driven by lesion size (Mattioli et al., 2014), but more likely by lesion location and load: For example, the structural integrity of subcortical white matter tracts (i.e., the arcuate fasciculus, Marchina et al., 2011) and specific left hemispheric cortical regions have been identified to influence recovery (Fridriksson, 2010; Bonilha et al., 2016). Moreover, non-patient related factors (e.g., therapy method, intensity, and language task used during neuroimaging) may strongly influence recovery. By focusing on intensive and effective interventions with chronic patients, the number of potential confounds of previous research might be diminished. To date, very few studies on neuroplastic changes following intensive, successful SLT have been published. These studies will be focused on now: Intensive aphasia therapy (a minimum of 1–2 h/d, for at least 2 weeks, Bhogal et al., 2003) combined with functional or structural neuroimaging in chronic PSA patients has been reported for three different methods. The first method, *Constraint-Induced Aphasia Therapy* (CIAT, Pulvermüller et al., 2001), also called *Intensive Language Action Therapy* (ILAT, Difrancesco et al., 2012) is one of the most researched SLTs and its clinical effectiveness has been demonstrated in several RCTs (Pulvermüller et al., 2001; Meinzer et al., 2005; Berthier et al., 2009; Stahl et al., 2016). The other two intensive therapy regimes, *anomia treatment* (Bonilha et al., 2016) and *melodic intonation therapy* (MIT, Schlaug et al., 2009) have shown to be effective, as evidenced by group studies or case series.

CIAT/ILAT-induced changes in brain activation following 2 weeks of intensive training (3 h/d) were observed using various neuroimaging methods and language tasks. A significant body of data was obtained with EEG and MEG and language tasks applied comprised lexical decisions (Pulvermüller et al., 2005), passive reading of words (Barbancho et al., 2015) and an auditory passive listening mismatch negativity (MMN, Näätänen et al., 1997) design (Lucchese et al., 2016; Mohr et al., 2016). The source estimates performed on EEG lexical decision data suggested activation changes in right-frontal and left-temporoparietal areas correlating with clinical language improvements (Pulvermüller et al., 2005). In an fMRI study, pre-post-therapy changes in neurometabolic brain activation brought about by ILAT were reported in RH frontotemporal areas when patients were auditorily processing complex and semantically ambiguous sentences (Mohr et al., 2014). A previous fMRI study (Meinzer et al., 2008) found ILAT-induced metabolic changes in LH perilesional areas during an overt naming task. Similarly, in an MEG study, ILAT led to enhancement of LH perilesional neuromagnetic activity evoked by words presented in a passive auditory MMN paradigm and which correlated with clinical improvements (Mohr et al., 2016).

Although these intensive short-term studies were not RCTs, the results of several of them can be interpreted, because significant correlations between clinical language improvement and brain activation changes were found. As this therapy method uses communicative-pragmatic language training, involvement of residual language regions and neuroplasticity in both hemispheres can be assumed. Still, the diversity of results across studies of ILAT shows that the *language task* and *stimulus type* might be important factors that could influence the topography of neuroplastic changes.

While bi-hemispheric neural recruitment and improvements of naming had previously been reported in short-term, intensive *anomia treatment* for trained items only (Fridriksson et al., 2006), recent studies found improved naming, and thus generalization effects, also for untrained items (Fridriksson et al., 2012). However, as this treatment method specifically focuses on naming, changes in other language domains were not reported. Neuroplastic changes following anomia treatment showed an increase of brain activation during picture naming which was observed in fronto-temporal LH perilesional areas with fMRI (Fridriksson et al., 2010, 2012). These activity changes across the 2 weeks of intensive training (also 3 h/d) correlated with improvements in naming performance, which were associated with intact functional connections between preserved cortical areas of the language network that were responsive to therapy (Bonilha et al., 2016). Anomia therapy involves training of overt naming of concrete objects depicted on cards by using either semantic or phonological cueing and thus, focuses on one specific aspect of aphasia, namely anomia and linguistic naming excercises, but does not emphasize behavioral relevance or communicative use of language. Consequently, training-induced neuroplasticity may specifically tap into left-hemispheric residual language networks associated with the abilitiy to name objects.

Using structural DTI before and after intensive *melodic intonation therapy* (MIT), an increase in fiber density and volume of the arcuate fasciculus in the RH was reported (Schlaug et al., 2009). However, as no correlation was found between language improvement and structural brain changes after therapy, it is difficult to interpret these structural data. Interestingly, in another study by this group, improvements in speech production after MIT correlated with structural changes in right hemisphere inferior frontal gyrus in patients with Broca's aphasia (Wan et al., 2014). Language improvement in spontaneous speech and re-structuring of the language system in the RH after MIT could be driven by aspects of this therapy method which possibly involve right-hemisphere dominant cognitive and motor functions such as melodic intonation, rhythm, and left hand tapping (Schlaug et al., 2009). It should be noted though, that while treatment intensity and duration in ILAT/CIAT and anomia treatment studies were completely identical (3 h/d for 2 weeks), MIT was applied less intensively (1.5 h/d), but with longer duration (~16 weeks), resulting in approximately three times higher amounts of overall treatment for MIT than for the other two therapies.

Overall, evidence suggests that intensive aphasia treatment in chronic PSA leads to reorganization of the functional and structural language network in both hemispheres. The involvement of each hemisphere in neuroplasticity is probably independent from the neuroimaging method used, but may be strongly influenced by the *therapy method, its intensity, duration*, and the *language materials and tasks*.

## The therapeutic method and the language tasks influence neuroplastic changes in the left and right hemisphere

In chronic PSA, the neuronal processes underlying training-induced language re-learning may involve re-activation of residual language networks in the LH and/or increased activation of spared and still intact parts of the RH language network. These areas may be primarily involved in re-establishing synaptic contacts within (partially) spared structural regions and lexico-semantic language circuits in both hemispheres. On this basis, increased post-treatment brain activation which correlates with improved language and communication abilities can be interpreted to reflect a more efficient and partially re-established language network. The type of task used to assess language processing during neuroimaging is likely to have a major impact on the functional lateralization of brain activation: overt picture naming and automatic auditory processing of words seem to recruit LH perilesional areas while recruitment of both hemispheres were reported for tasks involving complex lexico-semantic comprehension, passive reading of words and lexical decision. Thus, choosing appropriate language tasks is essential when investigating changes in brain activation: active language tasks (e.g., naming, lexical decision) imply sustained attention and behavioral responses, making it difficult to disentangle brain activity associated with language processing from motor, attentional, or other cognitive processes. Furthermore, active tasks are often effortful, particularly for patients with non-fluent PSA due to their speech production and motor problems, often resulting in high error rates. Brain activation measured using complex, effortful tasks could, at least in part, reflect error monitoring or cognitive strategies rather than language processing *per se*. In contrast, auditory perception and comprehension abilities are often relatively well-preserved in non-fluent PSA patients, therefore, passive auditory language tasks (e.g., silent reading, MMN) may be better suited to map language reorganization, particularly in this specific patient group. For example, the MMN is an experimental design where participants are not required to attend to stimuli and are in fact instructed to focus their attention on a distracting and entertaining film. The MMN is known to be well-suited for the investigation of patient populations who may have difficulties in maintaining attention, performing a specific task, or executing motor movements (Näätänen et al., 2012). Thus, the experimental features of the MMN design seem well-suited for studying neuroplasticity in non-fluent PSA and can be applied even in severely affected patients.

## Conclusion

Intensive, effective, and short-term aphasia therapy methods are promising and well-suited to investigate language recovery and neuroplasticity. Perilesional areas in the LH as well as fronto-temporal regions in the RH seem to contribute to therapy-induced neuroplasticity. Neuronal changes in both hemispheres may depend on the lesion load of subcortical white matter tracts as well as on the structural integrity of preserved left-hemispheric cortical regions which are part of the premorbid language network. The degree of hemispheric involvement is likely to be influenced by the treatment method, language materials, and task applied during neuroimaging. In this context, the use of passive language tasks seems advantageous when mapping language-related functional changes, particularly in patients with non-fluent aphasia.

In future, randomized controlled intervention studies will be essential to identify relevant predictors of recovery, to determine the suitability of patients for specific interventions and finally, to better understand the functional connection between language recovery and associated brain plasticity in patients with chronic post stroke aphasia.

## Author contributions

The author confirms being the sole contributor of this work and approved it for publication.

### Conflict of interest statement

The author declares that the research was conducted in the absence of any commercial or financial relationships that could be construed as a potential conflict of interest.
